# Editorials

**Published:** 1910-08

**Authors:** 


### EDITORIALS

The Business Office of the *JOURNAL-RECORD* is 1 1-2 to 5 1-2 South Broad Street.
The Editorial Office is 1014-15 Century Building.
Address all Business Communications to *Journal-Record of Medicine*, 1 1-2 to 5 1-2 South Broad Street.
Make remittances payable to *THE JOURNAL-RECORD OF MEDICINE.*
On matters pertaining to the Editorial and Original Communications, address Edgar G. Ballenger, M. D., Atlanta, Ga.
Reprints of Original Articles will be furnished at cost price. Requests for the same should always be made in THE MANUSCRIPT.
We will present, postpaid, on request, to each contributor of an original article, twenty (20) marked copies of *THE JOURNAL-RECORD OF MEDICINE* containing such article.

### TO THOSE ABOUT TO MARRY.

Regarding the recent agitation as to how a girl can be assured that her prospective husband is free from disease, *Judge*, in the number for July 23, makes the suggestion to women about to marry and that is, not to marry "unless the groom has an insurance policy in a reliable company." This advice is good but even better is the awakening of the public as to the necessity of such a health certificate. *Judge* lays especial stress upon the character of the company. This, of course, is an important factor but not of so great importance as is the date of the policy. No matter how excellent the company, it cannot prevent the insured from becoming infected with gonorrhoea or syphilis after the policy has been issued. If the policy is of recent date, it should lessen the danger to the bride-to-be; such a custom would place her on guard and show her parents how reasonable assurance of health might be obtained without insult or insinuation. Unfortunately many of the poorer classes who are subjected to the greatest dangers are not able to procure a life insurance policy; the *necessity* of such a "certificate of health" would, however, be productive of good.

This kind of recognition of danger will ultimately lead legislators to pass laws that are reasonable and yet effective, at least in part.

### "PHARMACOLOGIC FETISHISMS."

With "Pharmacologic Fetishisms" a sits title, Barton, in *The Journal of the American Medical Association,* July 23, 1910, makes an attack on a number of well-known drugs, such as calomel, aconite, ammonium chloride, sweet spirits of nitre, viratrum viride, etc. The matter seems to have been considered exclusively from the standpoint of annual experimentation or at least one would judge so from the nihilistic views expressed. So at variance are Barton's ideas with those of thousands of practitions who are daily using these remedies, that a protest hardly seems necessary. Whether calomel acts as a cholegogue or as an intestinal antiseptic matters not at all to the patient with intestinal auto-intoxication or the so-called biliousness. Incontestable facts have proved calomel to be the best remedy to make the patient well under such circumstances, so why "split hairs" and bewilder the physician who already has few enough of drugs with physiologic effects that may be relied upon? Have we not all seen the diuretic effect of sweet spirits of nitre? Are there sufficient millions of animals to prove it has no such action in human beings who are being treated with it? Spartem is maligned as being a worthless cardiac stimulant, but not a word said of its value as a renal stimulant; given hypodermically in 1 to 2 grain doses, the writer knows of no more dependable drug when suppression of the urine is feared—especially if it be given early, for instance, 12 hours before an operation where suppression is a danger.

It is not to fight the individual battles of these separate drugs that this editorial is written, but rather is it intended to condemn the indiscriminate nihilism underlying the above article. For the sake of hyper-scientific accuracy, Barton marshals before us an array of petty points of weakness of our most valuable drugs, and thus he weakens our faith in our best and most reliable friends and makes us all the easier prey to the alleged virtues of many worthless proprietary preparations so scientifically (?) heralded before us. If we are not to use these drugs which are condemned, in part if not totally, then what *should* we use in their stead? Can Barton provide for us more reliable drugs than those he declares so worthless?

---

### EHRLICH'S NEW REMEDY FOR SYPHILIS.

If the preliminary reports are substantiated by further observation and more extended trial, Ehrlich and Hata will have contributed to the medical profession perhaps its most remarkable drug. For considerable time, Ehrlich has been experimenting with the arsenical preparations hoping to find one which would have a specific anti-spirochetal power and it seems that in his remedy "606" (dimethyl-dioxiarsenobensoldichlorhvdrat) he has succeeded. At least, Germany seems quite wild over the success so far attained. At the Berlin Medical Society, June 22, 1910, Wechselmann, to whom Ehrlich entrusted the clinical tests of his remedy at the Virchow Hospital, reported that the new drug acted on the symptoms of syphilis, in all of their infectious forms, with a rapidity and thoroughness which cannot be approached by any other known remedy. He has tested it, in eighty cases, and states that its beneficial action occurred with the certainty of an experiment. Ehrlich claims that "one injection utterly destroys all the sprichoetes, but is harmless to animal tissues." The dose varies from 5 to 10 grains intramuscularly and is followed by considerable rise of temperature and pain at the seat of the injection. Neisser says that it is yet too early for a definite opinion, but that it "can be affirmed with certainty that the new remedy exerts a remarkable, even surprising action on the spirochetes, as well as the syphilitic products themselves. Spirochetes disappear in very many cases after from twenty-four to forty-eight hours from primary lesions and condylomata in which they were abundantly present before administration."

Schreiber, Hoppe, Loeb, Treupel, Fischer and others have made favorable reports of patients treated with this preparation. Schreiber and Hoppe administered it intravenously in thirty cases and found the rise of temperature and the unpleasant side effects did not occur. In fifty-two cases they found that the Wassermann reaction became negative in 92.3 per cent. This occurred in the earliest case in four days, and in the latest, seventy days after injection. In most cases this occurs in 14 days.

Ehrlich declines to place his remedy in the hands of private practitioners until 3,000 cases have been reported on; as 2,500 phials of the remedy have already been distributed to physicians upon the agreement in writing that a report of the results will be sent to him, definite facts will soon be known. It seems "to good to be true" and until the final report is made we will remain conservative, but open to conviction when proof is presented.

---

### ILLNESS OF DR. J. P. WATKINS.

With deep regret we have heard of the critical illness of Dr. J. P. Watkins, president of the Board of Councillors of the Chattahoochee Valley Medical Association.

